# Monsef Benkirane awarded 2013 Ming K. Jeang Foundation Retrovirology Prize: Landmark HIV-1 research honoured

**DOI:** 10.1186/1742-4690-10-38

**Published:** 2013-04-05

**Authors:** Ben Berkhout, Andrew Lever, Mark Wainberg, Ariberto Fassati, Persephone Borrow, Masahiro Fujii

**Affiliations:** 1 Department of Medical Microbiology, Laboratory of Experimental Virology, Center for Infection and Immunity Amsterdam (CINIMA), Academic Medical Center, University of Amsterdam, Meibergdreef 15, Amsterdam, 1105 AZ, The Netherlands; 2 Department of Medicine, University of Cambridge, Addenbrooke’s Hospital, Cambridge, CB2 0QQ,, UK; 3 McGill University AIDS Centre, Lady Davis Institute, Jewish General Hospital, Montreal, Québec, Canada; 4 The Wohl Virion Centre and MRC Centre for Medical & Molecular Virology, Division of Infection and Immunity, University College London, Cruciform Building, 90 Gower Street, London, WC1E 6BT,, UK; 5 Nuffield Department of Clinical Medicine, University of Oxford, Weatherall Institute of Molecular Medicine, John Radcliffe Hospital, Headington, Oxford,, OX3 9DS,, UK; 6 Division of Virology, Niigata University Graduate School of Medical and Dental Sciences, Niigata, Japan

## 

Dr. Monsef Benkirane, from the Laboratoire de Virologie Moléculaire in Montpellier, France, has been announced as the recipient of the 2013 Retrovirology Prize. This bi-annual prize covers all aspects of the Retrovirology field and celebrates groundbreaking research from retrovirologists aged between 45 and 60. Monsef is among the brightest young “stars” in HIV-1 biology. At 45 years of age, he is the youngest recipient of the Retrovirology prize. This year the competition was particularly fierce with 4 strong contenders.

Monsef received training in immunology at the University of Marseille, France, and came to the laboratory of our former editor-in-chief Kuan-Teh Jeang at the NIH in 1995, where he worked on HIV-1 transcription and post transcriptional regulation. In 1998, Monsef established his own laboratory at the Human Genetics Institute in Montpellier, France. He published the first paper showing that the transcriptional activity of Tat is regulated by post-translational modifications such as acetylation and ubiquitylation [1,2]. He also published a pioneering study on the identification of the subunit composition of the Positive Transcription Elongation Factor, PTEFb.

He focused on understanding the molecular mechanisms leading to the establishment and maintenance of transcriptionally silent HIV provirus, an important area of research for the design of virus eradication strategies (Cure for AIDS) [3]. His team provided the first demonstration of the role of the histone methyltransferase Suv39H1 in HIV-1 transcriptional silencing [4]. Additionally, he unraveled an intricate crosstalk between HIV transcription and the cellular RNAi machinery [5-7].

Most recently, Monsef has worked on a puzzle that has occupied the study of HIV-1 for over 2 decades: why myeloid cell and quiescent CD4+ T cells are refractory to HIV-1 replication. He and his team have made ground breaking contributions in this field. They identified SAMHD1, a protein encoded by an Aicardi-Goutières Syndrome susceptibility gene, as the HIV-1 restriction factor operating in dendritic cells, macrophages and quiescent CD4+ T cells [8]. The identification of the restriction factor operating in these cells has been a very challenging and competitive area of research that received quite some attention in our journal recently [9-11].

Dr. Benkirane’s honors in research recognition include elected membership in EMBO and French Academy of Science Award (both 2012), and he was recently elected to the American Academy of Microbiology (2013). He has published over 60 peer-reviewed manuscripts and review articles, very often in top journals such as Cell and Nature. He has been an Editor of Retrovirology since 2004 and organized the first Frontiers of Retrovirology meeting in Montpellier in 2009.

To mark the award, an editorial will be published in BioMed Central's open access journal *Retrovirology*. The Retrovirology Prize recipient is selected by *Retrovirology*'s Editors based on nominations submitted by the journal's Editorial Board. The biennial prize for HIV or non-HIV related research consists of a $3,000 check and a crystal trophy. He will receive the prize at the upcoming third Frontiers of Retrovirology meeting in Cambridge, UK (http://www.frontiers-of-retrovirology.com).

Past winners include Professor Masao Matsuoka from Japan (2011), Professor Mike Malim from the United Kingdom (2010) and Professor Thierry Heidmann from France (2009).
